# Changes in clinical management of diffuse *IDH*-mutated lower-grade gliomas: patterns of care in a 15-year period

**DOI:** 10.1007/s11060-022-04136-y

**Published:** 2022-11-25

**Authors:** Caroline Svenjeby, Louise Carstam, Katja Werlenius, Thomas Olsson Bontell, Isabelle Rydén, Julia Jacobsson, Anna Dénes, Asgeir S. Jakola, Alba Corell

**Affiliations:** 1 Institute of Neuroscience and Physiology, Department of Clinical Neuroscience, Sahlgrenska Academy, University of Gothenburg, Gothenburg, Sweden; 2 Department of Neurosurgery, Sahlgrenska University Hospital, Gothenburg, Sweden; 3 Department of Oncology, Sahlgrenska University Hospital, Gothenburg, Sweden; 4 Department of Oncology, Institute of Clinical Sciences, Sahlgrenska Academy, University of Gothenburg, Gothenburg, Sweden; 5 Department of Physiology, Institute of Neuroscience and Physiology, Sahlgrenska Academy, University of Gothenburg, Gothenburg, Sweden; 6 Department of Clinical Pathology, Sahlgrenska University Hospital, Gothenburg, Sweden

**Keywords:** Low-grade glioma, Treatment outcome, Brain neoplasm, Neurosurgery

## Abstract

**Background:**

*Isocitrate dehydrogenase* (*IDH*) mutated diffuse lower-grade gliomas (dLGG) are infiltrating brain tumors and increasing evidence is in favor of early multimodal treatment. In a Scandinavian population-based setting, we wanted to study treatment patterns over the last 15 years, focusing on the short-term postoperative course to better understand the potential negative consequences of treatment.

**Methods:**

Patients ≥ 18 years with primary *IDH*-mutated dLGG grade 2 and 3, operated between January 2007–June 2021 were identified. Patients were divided into subgroups (2007–2011, 2012–2016, and 2017–2021) and comparisons regarding tumor- and disease characteristics, treatment, and postoperative outcome were performed.

**Results:**

We identified 202 patients (n = 61, 2007–2011; n = 72, 2012–2016; n = 69, 2017–2021), where of 193 underwent resection without change in proportion of resections over time. More patients underwent complete resections in recent times (6.1%; 15.7%; 26.1%, respectively; p = 0.016). Forty-two patients had any neurological deficit postoperatively (14.8%; 23.6%; 23.2%; p = 0.379), mostly minor and transient. Differences in oncological therapy were seen between the investigated subgroups. Early radiotherapy alone (32.8%; 7%; 2.9%; p < 0.001), concomitant chemoradiotherapy (23%; 37.5%; 17.4%; p = 0.022), sequential chemoradiotherapy (0%; 18%; 49.3%; p < 0.001), and no adjuvant treatment (42.6%; 23.6%; 18.8%; p = 0.009) shifted during the studied period. Increasingly more patients received proton radiotherapy compared to photon radiotherapy during the later time periods (p < 0.001).

**Conclusion:**

Complete resections were performed more often in later time periods without an apparent increase in surgical morbidity. Early adjuvant oncological treatment shifted towards providing chemotherapy and combined chemoradiotherapy more often in later time periods. Protons replaced photons as the radiation modality of choice.

**Supplementary Information:**

The online version contains supplementary material available at 10.1007/s11060-022-04136-y.

## Introduction

Diffuse lower-grade gliomas (dLGGs) are slow-growing infiltrative tumors, typically affecting adults of younger age [1]. Over time, accumulating molecular aberrations tend to cause a more malignant tumor, leading to increased morbidity and premature death. The estimated overall survival (OS) is highly dependent on individual risk factors and the biological profile of the tumor. Recently, the 2021 version of the World Health Organization (WHO) Classification of tumors in the central nervous system further emphasized the molecular aspects of the classification, where adult-type dLGGs are characterized by the *IDH*-mutation [2] Moreover, due to the controversy of WHO-grading within the *IDH*-mutated tumors, it is now more common to report diffuse grade 2 and 3 astrocytomas (*IDH-*mutant without 1p19q codeletion) and oligodendrogliomas (*IDH-*mutant with 1p19q-codeletion) together under the term lower-grade gliomas [3,4].

There have been several important papers demonstrating that more active treatment of *IDH*-mutated tumors significantly prolongs survival [5–7]. Resection compared to biopsy is the preferred surgical strategy and a dose-response relationship is present, where a higher extent of resection (EoR) is favorable [7–10]. The adjuvant treatment strategy has changed in recent years following landmark papers demonstrating a clear survival benefit of the addition of upfront chemotherapy (procarbazine, lomustine, and vincristine, PCV) to radiotherapy [11–14] .

Transient neurologic deterioration following surgery is common [15–18]. A more active surgical strategy logically puts the patient at an increased neurological risk. For oncological therapy, perceived high-risk patients with *IDH*-mutated gliomas, according to the studies by Pignatti et al. [19] and Buckner et al. [12] from the pre-molecular era, are often given adjuvant treatment upfront following surgery to minimize the risk for rapid tumor progression and relapse. The perceived low-risk patients, on the other hand, are often managed with wait-and-scan to minimize the risk of long-term consequences of radiotherapy [20,21].

Altogether, it is likely that there has been a change during the past decade in the management of patients with *IDH*-mutated dLGG. We presumed a more active surgical approach and a higher proportion of patients receiving upfront oncological treatment to be found. Specifically, for the surgical treatment, we hypothesized that more patients in recent time periods underwent resection, and for those resected, a higher EoR was anticipated.

## Materials and methods

### Patient cohort

All patients in the western health care region of Sweden with newly diagnosed suspected primary intracranial intraaxial tumors, are managed by a multidisciplinary team (MDT) with weekly conferences at the Sahlgrenska University Hospital, Gothenburg, Sweden.

We aimed to include all patients fulfilling the study criteria in a database at Sahlgrenska University Hospital, during a time spanning from January 2007 to June 2021. Inclusion criteria were all patients ≥ 18 years and a primary histological diagnosis of *IDH*-mutated dLGG grade 2 and 3 during the study period. Patients with IDH mutated grade 4 astrocytomas were excluded. Patients were divided into three subgroups based on balanced time periods of histopathological diagnosis (2007–2011, 2012–2016, 2017–2021) before data analysis, taking into consideration those landmark papers by Jakola et al. [22] and Buckner et al. [12] could have affected the treatment strategy during this time in a surgical and oncological aspect, respectively [12,23]. These time periods were based mainly on these two papers due to their perceived clinical impact and publication year, 2012 respectively 2016, to evaluate the subsequent clinical effects. In total, 202 patients were identified. Data was collected in a retrospective fashion and all patients were followed at least six months after surgery.

### Clinical and radiological characteristics

The demographic, surgical, and clinical data was retrospectively collected from electronic health records. Preoperative Karnofsky performance status (KPS) score was divided into three groups; 80–100 (normal activity), 50–70 (care for self with occasional assistance but unable to work or perform normal activities), 0–40 (patients with significant care needs, including hospitalization or institutionalization) [24]. Preoperative deficits were recorded from the patients logs. Cognitive deficit recorded was based on patient-reported symptoms and not necessarily cognitive assessment, and recorded from patient logs. Severe cognitive deficits affected the patient’s ability to work and daily life. The cognitive deficit was not based on the objective neurophysiologic assessment, as this was not routine during the first and second period. Post-operative neurological deficits were categorized as either any new or worsened deficit after surgery, with further classification into either minor or severe ones. Severe deficits were defined as aphasia or severe dysphasia with an impact on effective communication, hemianopia, motor function grade < 4 on the Medical Research Council (MRC) scale based on patient logs or cognitive deficit affecting daily life [18,25]. Deficits persisting more than 3 months were considered permanent [18]. Neurosurgical complications were evaluated using the Landriel-Ibanez classification within 30 days of surgery, [26] and neurological deficits were presented separately.

The surgical techniques were collected from the operation logs and included type of surgery (resection or biopsy), use of neuronavigation, ultrasound, motor mapping and monitoring under general anesthesia, awake mapping, ultrasonic aspirator and use of 5-ALA florescence. 5-ALA was used for hotspot detection and guided by regular microscope.

Type of radiation (photons or protons), and chemotherapy were registered. In this study, focusing on the initial management, adjuvant oncological treatment was defined as having chemotherapy and/or radiotherapy within six months after surgery. The patient was registered to have no oncological treatment if none was started within six months from surgery. In order not to miss planned sequential treatments started prior to six months, all patients who received oncological treatment were followed up to 12 months after surgery where both modalities were registered even if only one of them was initiated prior to six months. Chemoradiotherapy was defined as having either concomitant or sequential treatment.

Tumor volume was assessed by segmentation on magnetic resonance imaging (MRI) scans on the sequence where the glioma was best visualized (T2w or FLAIR sequences) with the software 3D slicer version 4.6.2 in accordance with previously published studies [27,28]. The tumor volume measurements were performed for general research purposes (not this specific project) and no specific measures were taken for blinding of clinical outcome and histomolecular data. The volumetric analysis was performed by several trained researchers and research assistants, and all were validated by an experienced neurosurgeon (A.S.J.) The postoperative volumes were calculated on MRI scans < 4 months of surgery. For some patients with postoperative scans performed > 4 months, it was possible to make a qualitative assessment regarding the variable complete versus partial resection, but cases with postoperative MRI performed later than 3 months after surgery were not included for volumetric assessment, but with the exemption being patients with radical resection where the volume was considered to be 0 cm [3].

Routine clinical analysis of *IDH*-status using a step-wise approach with initial IHC for R132H mutation followed by next-generation sequencing for *IDH1* and *IDH2* mutations if negative IHC was introduced in 2016. Patients diagnosed prior to 2016 have been reclassified as described in previous work [29].

### Statistical analysis

IBM SPSS Statistics software program version 28.0 was used to perform statistical analyses. Data were presented as count values, proportions in percentage, means, and median, and the spread was presented with SD or quartiles one and three (Q1, Q3). The three subgroups were compared regarding categorical data using crosstabs and Pearson’s chi-square test or Fisher’s exact test (Fisher-Freeman-Halton), with a significance level set to 0.05. The distribution of continuous data were assessed by using Q-Q plots. Normally distributed data were analyzed with ANOVA, otherwise with Kruskal-Wallis.

## Results

### Baseline characteristics

A total of 202 patients were identified: 61 patients operated between 2007 and 2011 (Group 1), 72 patients between 2012 and 2016 (Group 2), and 69 patients between 2017 and 2021 (Group 3). The mean age at surgery was 41.7, 42.5, and 45.3 years in each group, respectively. Females were underrepresented in all three groups. Seizure was the most common presenting symptom. A detailed description of baseline characteristics is presented in Table 1. The preoperative and postoperative median tumor volumes were similar over time, and did not statistically differ between groups preoperatively (p = 0.329) and postoperatively (p = 0.401). However, complete resections increased over time from 3 patients (6.1%) in the earliest group to 18 patients (26.1%) in the latest period (p = 0.016).

**Table 1:** Baseline characteristics in patients with diffuse *IDH-*mutated lower-grade glioma WHO grade 2 and 3, n = 202

	Group 1 (2007–2011)	Group 2 (2012–2016)	Group 3	P-value
	n = 61	n = 72	(2017–2021)	
			n = 69	
Age (years), mean (SD)	41.7 (12.8)	42.5 (12.5)	45.3 (14.3)	0.27
Female, n (%)	25 (41.0)	30 (41.7)	28 (40.6)	0.991
**Symptoms at presentation, n (%)**				
Asymptomatic	4 (6.6)	7 (9.7)	7 (10.1)	0.784
Seizure	45 (73.8)	56 (77.8)	44 (63.8)	0.167
Headache	10 (16.4)	10 (13.9)	13 (18.8)	0.729
ICP-related symptoms	11 (18.0)	12 (16.7)	12 (17.4)	0.979
Any deficit	11 (18.0)	9 (12.5)	17 (24.6)	0.176
- Motor deficit	7 (11.5)	4 (5.6)	6 (8.7)	0.459
- Language deficit	3 (4.9)	5 (6.9)	7 (10.1)	0.526
- Cognitive deficit	8 (13.1)	8 (11.1)	12 (17.4)	0.602
- Visual deficit	3 (4.9)	1 (1.4)	6 (8.7)	0.118
**Karnofsky performance score, n (%)**				0.085
80–100	43 (70.5)	60 (83.3)	55 (79.7)	
50–70	18 (29.5)	11 (15.3)	11 (16.0)	
0–40	0 (0.0)	1 (1.4)	3 (4.3)	
Pre- and/or postoperative neuropsychologic assessment, n (%)*				
	N = 58	N = 72	N = 69	
Both pre- and postop	2 (3.5)	15 (20.8)	49 (71.0)	**< 0.001**
Preop only	1 (1.7)	0 (0.0)	2 (3.0)	0.396
Postop only	8 (13.8)	14 (19.5)	9 (13.0)	0.551
No	47 (81.0)	43 (59.7)	9 (13.0)	**< 0.001**
**Radiological evaluation, n (%)**				
- fMRI	13 (21.3)	21 (29.2)	17 (24.6)	0.577
- DTI/tractography	1 (1.6)	17 (23.6)	9 (13.0)	**< 0.001**
- Perfusion MRI	4 (6.6)	21 (29.2)	26 (37.7)	**< 0.001**
- MR spectroscopy	1 (1.6)	3 (4.2)	17 (24.6)	**< 0.001**
- FET-PET	0 (0.0)	0 (0.0)	2 (2.9)	0.206
- Navigated TMS	1 (1.6)	17 (23.6)	11 (15.9)	**< 0.001**
**Main location and larges diameter, n (%)**				0.174
Frontal	39 (63.9)	47 (65.3)	38 (55.1)	0.409
Temporal	12 (19.7)	15 (20.8)	13 (18.9)	0.956
Insular	2 (3.3)	3 (4.2)	9 (13.0)	**0.047**
Parietal	6 (9.8)	7 (9.7)	9 (13.0)	0.779
Occipital	2 (3.3)	0 (0.0)	0 (0.0)	0.097
Bilateral, n (%)	9 (14.8)	7 (9.7)	6 (8.7)	0.558
Multifocal, n (%)	1 (1.6)	3 (4.2)	3 (4.3)	0.786
Largest diameter (mm), mean (SD)	58.1 (18.4)	54.7 (19.8)	55.4 (20.4)	0.588
**Histological assessment, WHO 2016, n (%)**				
Astrocytoma	35 (57.4)	41 (56.9)	33 (47.8)	
- Grade 2	16 (45.7)	17 (41.5)	25 (75.8)	**0.007**
- Grade 3	19 (54.3)	24 (58.5)	8 (24.2)	**0.007**
Oligodendroglioma	26 (42.6)	31 (43.1)	36 (52.2)	
- Grade 2	13 (50.0)	18 (58.1)	16 (44.4)	0.538
- Grade 3	13 (50.0)	13 (41.9)	20 (55.6)	0.538
Segmentation volume (mL), median (Q1, Q3)*				
Preoperative	N = 60	N = 71	N = 69	0.329
	54.8	55.4	45.1	
	(26.6, 119.2),	(30.0, 87.1),	(22.3, 88.5)	
Postoperative	N = 30	N = 70	N = 69	0.401
	8	11.2	3.4	
	(2.6, 47.9),	(1.7, 36.7),	(0.0, 21.0)	
Gross total resection, n (%)	N = 49	N = 70	N = 69	**0.016**
	3 (6.1)	11 (15.7)	18 (26.1)	

Abbreviations: DTI, diffusion tensor imaging; ICP, intracranial pressure; KPS; mL, milliliter; mm, millimeter; MRI, magnetic resonance imaging; PET, positron emission tomography; postop, postoperatively; preop, preoperatively; Q1, 25th percentile; Q3, 75th percentile. SD, standard deviation; TMS, transcranial magnetic stimulation; WHO, world health organization. *Indicates that values are missing, see actual N provided per cell

Most patients had a KPS score between 80 and 100. Over time, a larger number of patients underwent neuropsychologic assessment both pre- and postoperatively. DTI/tractography, perfusion MRI, MR spectroscopy, and navigated TMS were performed to a greater extent in Groups 2 and 3. Most dLGGs were unifocal, unilateral, and located in the frontal or temporal lobe.

### Treatment variables and techniques

As seen in Table 2, 193 patients in total were treated with resection, compared to nine patients that underwent biopsy only and there was no change in the ratio of resected versus biopsied patients over time (p = 0.977). The proportion of complete resections increased over time (2007–2011; 6.1%, 2012–2016; 15.7%, and 2017–2021; 26.1%; p = 0.016), see Table 1. Eight patients in total underwent resection supported by 5-ALA (0%, 8.7%, and 3%, p = 0.073) and nine patients, all of whom in group 3 had intraoperative MRI (13.6% of patients in Group 3).

**Table 2:** Treatment variables and surgical tools

	Group 1	Group 2	Group 3	P-value
	(2007–2011)	(2012–2016)	(2017–2021)	
	n = 61	n = 72	n = 69	
**Initial surgical procedure**				
Resection, n (%)	58 (95.1)	69 (95.8)	66 (95.7)	0.977
Biopsy, n (%)	3 (4.9)	3 (4.2)	3 (4.3)	
**Surgical technique in tumor resection**				
- Neuronavigation, n (%)	36 (63.2)	46 (66.7)	60 (90.9)	**0.002**
- Ultrasound, n (%)	18 (31.6)	42 (60.9)	50 (75.8)	**< 0.001**
- Motor mapping/monitoring asleep, n (%)	1 (1.8)	4 (5.8)	26 (39.4)	**< 0.001**
- Awake mapping, n (%)	0 (0.0)	16 (23.2)	15 (22.7)	**0.001**
- Ultrasonic aspirator, n (%)	35 (61.4)	43 (62.3)	30 (45.5)	0.147
- 5-ALA, n (%)	0 (0.0)	6 (8.7)	2 (3.0)	0.073

Abbreviations: MRI, magnetic resonance imaging

**Fig. 1:**
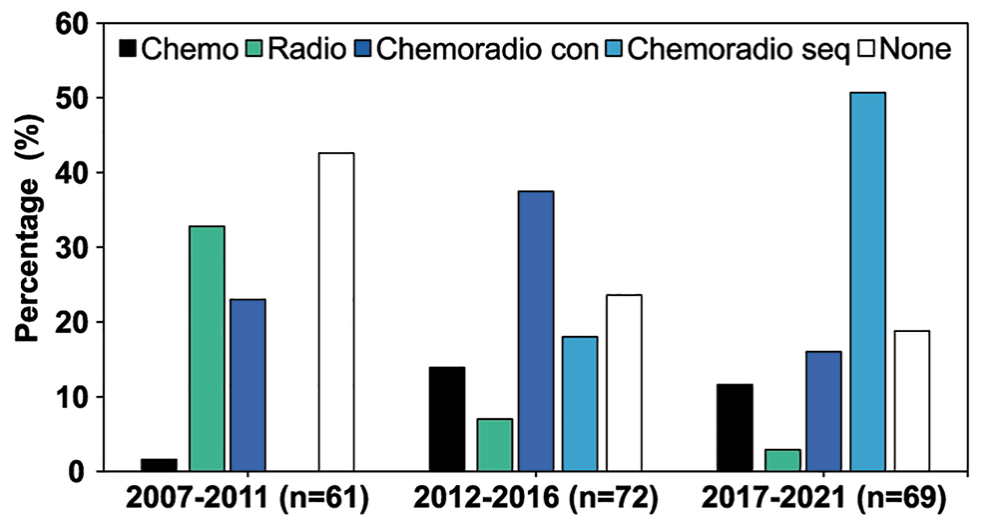
Adjuvant treatment following surgery. The bars represent percentage (%) of the total number of patients in each group. Chemo; chemotherapy alone. *Radio*; radiotherapy alone. *Chemoradio con*; concomitant chemoradiotherapy (e.g., radiotherapy concomitant with temozolomide +/- adjuvant). *Chemoradio seq*; sequential chemoradiotherapy (i.e. combined treatment but without concomitant chemotherapy). None; no adjuvant treatment.

The proportion receiving concomitant (p = 0.012) as well as sequential (p < 0.001) chemoradiotherapy within 12 months postoperatively (see details under Methods) increased significantly in the later groups. Likewise, the proportion of patients treated with chemotherapy alone after surgery was greater in Groups 2 and 3 (p = 0.022) as seen in Table 3; Fig. 1. Among patients receiving chemotherapy (either chemotherapy alone or chemoradiotherapy), the use of PCV increased over time (8.2%; 19.4%; 39.3%, p = 0.01), and consequently the use of Temozolomide (TMZ) decreased (91.8%; 80.6%; 57.1% p = < 0.001). All patients who had chemotherapy concomitant with radiotherapy received TMZ.

**Table 3:** Adjuvant treatment

	Group 1: 2007–2011 n = 61	Group 2: 2012–2016 n = 72	Group 3: 2017–2021 n = 69	P-value
Only chemotherapy^1^, n (%)	1 (1.6)	10 (13.9)	8 (11.6)	**0.022**
Only radiotherapy^1^, n (%)	20 (32.8)	5 (7.0)	2 (2.9)	**< 0.001**
Concomitant chemoradiotherapy^2^, n (%)	14 (23.0)	27 (37.5)	11 (16.0)	**0.012**
Sequential chemoradiotherapy^2^, n (%)	0 (0.0)	13 (18.0)	35 (50.7)	**< 0.001**
No adjuvant treatment^3^, n (%)	26 (42.6)	17 (23.6)	13 (18.8)	**0.009**

^1^Started within 6 months after surgery (see details under Methods)

^2^Within 12 months after surgery. Only TMZ was administered concomitant with radiotherapy

^3^Within 6 months after surgery

**Fig. 2:**
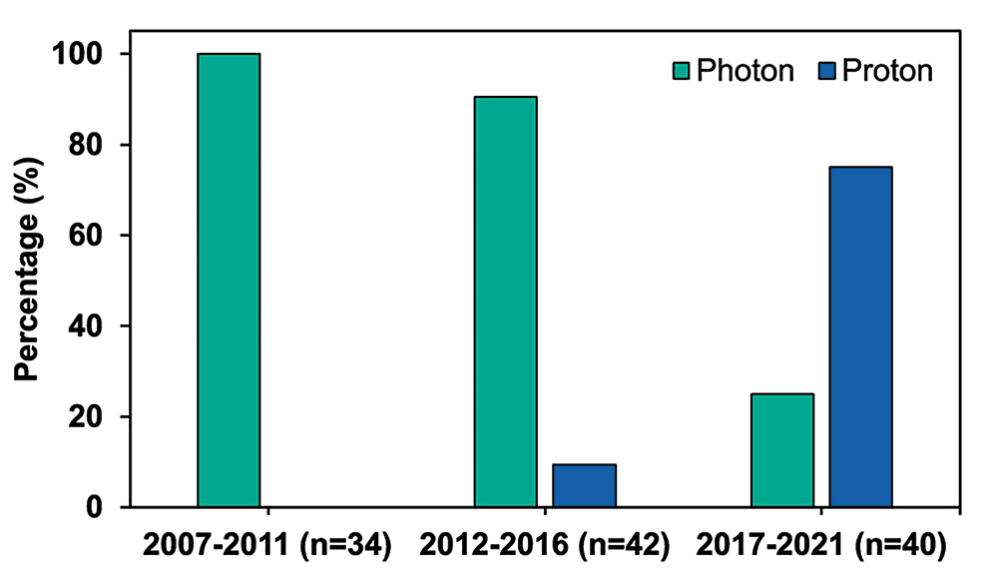
The bars represent percentage (%) of all patients treated with radiotherapy (radiotherapy alone or concomitant with chemotherapy) within 6 months after surgery, divided with respect to photon (100% 2007–2011, 90.5% 2012–2016, 25% 2017–2021) or proton (0%, 9.5%, 75%) radiotherapy

Administration of radiotherapy alone within six months postoperative was more common in the earliest group than in the later ones (p < 0.001), as shown in Fig. 1. Over time, the proportion of patients not receiving any adjuvant treatment within six months after surgery also decreased (p = 0.009). In irradiated patients, an increase in the proportion of patients receiving photon radiotherapy compared to proton radiotherapy was observed (p < 0.001) as seen in Fig. 2.

Only one patient received a hypofractionated course of radiotherapy. All other patients received radiotherapy at 1.8-2.0 Gy/fraction to a total dose of 50–54 Gy for grade 2 tumors or 59.4–60 Gy for grade 3 tumors, respectively.

### Postoperative complications and neurologic deficits

No complication after surgery according to the Ibanez classification was the most frequent outcome in all groups, with similar frequency over time as seen in Table 4. New or worsened neurological deficit postoperatively was common during all periods, also without any significant difference in incidence over time. Most of these patients suffered from nonsevere deficits as seen in Table 4 and Supplementary Tables 1 and 2.

**Table 4:** Postoperative complications and neurological deficits

	Group 1	Group 2	Group 3	P-value
	(2007–2011)	(2012–2016)	(2017–2021)	
	n = 61	n = 72	n = 69	
**Complications according to Ibanez classification, n (%)**				
	N = 59*	N = 72	N = 69	
No complication, n (%)	47 (79.7)	58 (80.6)	53 (76.8)	0.852
Grade < IIb	11 (18.6)	7 (9.7)	12 (17.4)	0.287
Grade ≥ IIb	1 (1.7)	7 (9.7)	4 (5.8)	0.156
**Neurological outcome postoperatively, n (%)**				
Patient with any deficit postoperativly, n (%)	22 (36.1)	30 (41.7)	32 (46.4)	0.492
- Motor (S / NS)	11 (1 / 10)	16 (4 / 12)	17 (3 / 14)	
- Language (S / NS)	9 (0 / 9)	16 (4 / 12)	13 (2 / 11)	
- Cognitive (S / NS)	9 (0 / 9)	6 (1 / 5)	11 (1 / 10)	
- Visual (S / NS)	2 (0 / 2)	7 (1 / 6)	4 (0 / 4)	
Patient with severe permanent deficit, n (%)	1 (1.6)	5 (6.9)	4 (5.8)	0.361
New or worsened seizure postoperatively, n (%)	0 (0.0)	2 (2.8)	5 (7.2)	0.073
Rehabilitation postoperatively, n (%)	4 (6.5)	13 (18.0)	11 (15.9)	0.115
30-day mortality, n (%)	0 (0.0)	0 (0.0)	0 (0.0)	

Abbreviations: postop, postoperatively; S, severe, NS, non-severe. * Indicates that values are missing, see actual N provided per cell

In total, 44 patients (14.8% in Group 1, 23.6% in Group 2, 26.1% in Group 3, p = 0.268) experienced any permanent deficit (i.e., any deficit, severe or nonsevere, persisting more than three months). Ten patients (5.0%) had a severe deficit persisting more than three months. For a detailed overview of permanent deficits, see Supplementary Tables 1 and 2. No patient died within 30 days. Table 4 presents additional details on complications and neurological deficits after surgery.

## Discussion

We found a higher rate of complete radiological resection in the later time periods without a significant increase in neurological morbidity. Furthermore, early oncological therapy has become more common with time, and there has been a shift towards the use of PCV instead of TMZ and proton radiotherapy instead of photon radiotherapy.

The proportion of patients having biopsy were considerably smaller than those having a resection, and a change in treatment pattern could not be seen over time. However, the number of complete resections increased over time, suggesting a more active surgical approach. The reasoning behind the more extensive surgery, although not analyzed specifically in this study, is that increased EoR has been shown to be strongly associated with improved survival, especially in patients with *IDH*-mutated astrocytomas [30,31].

Increased use of intraoperative tools such as mapping, monitoring, neuronavigation, and intraoperative imaging may indicate an attempt to achieve more radical resections, also in eloquent regions, which can come with a risk of more neurological impairment postoperatively. The majority of patients experienced no surgical complications, and no difference was seen over time. Regarding neurologic deterioration, temporary loss of function is a known risk after surgery, for instance, transient supplementary motor area (SMA) syndrome, which usually resolves within three months due to brain plasticity [32, 33]. There was a nonsignificant trend towards more neurologic deficits postoperative in recent time periods, although most were nonsevere and transient. The number of patients that experienced severe permanent neurologic deficits was small and the observed increase in recent time periods was not significant. We report a higher number of deficits compared to many other studies, but the reported proportion of permanent severe deficits (5.0%) aligns well with the reported deficits in the literature [18,34]. However, if this is due to a difference in reporting remains speculative. Furthermore, our definition of a permanent deficit (i.e., persisting more than three months) does not mean that further improvements are not seen in a longer perspective [35].

Neuropsychological assessment was more frequent in the later time periods. Since 2017, all patients with a presumed lower-grade tumor are assessed both pre- and postoperatively. Compared to earlier subjective evaluations of cognitive functioning, these assessments are more thorough and capture various aspects of cognitive functioning; visual and verbal learning and memory, perception and visuo-spatial abilities, verbal functioning, psychomotor speed, motor speed, executive functioning including inhibition, mental flexibility and planning, different aspects of attention, and working memory. The assessments also include measures of mental fatigue, quality of life, depression, and anxiety. Although not intentional, this more detailed assessment and functional focus may have affected both pre- and postoperative registration of neurological deficits. This may contribute to the observed nonsignificant increase in deficits over time. This speculation that our data is influenced by the increased awareness and discovery of cognitive deficits following 2017 is possibly further strengthened by the fact that in the latest period there were trends of patients who have smaller and incidental tumors, but still report a higher percentage of preoperative deficits.

We found that patients in the later time periods more frequently were treated with chemoradiotherapy, as well as with chemotherapy alone. The decreased use of TMZ in the later time periods could partially be related to fewer tumors with *IDH-*mutation being classified as astrocytoma grade 3. Receiving radiotherapy alone after surgery has become rare, 32.8% in the earliest time period to 2.9% in the last (p < 0.001), perhaps a clinical effect of the Buckner trial [12]. Over time, it has also become more common to receive adjuvant treatment, with only around 20% of patients being selected for wait-and-scan in the later groups. The consequences of early radiotherapy may not be noticeable early on as side effects may present years after treatment [21,36]. However, in patients with negative prognostic factors, the survival benefits outweighs potential late side effects [12,37]. Still, which parameters to be used as prognostic factors are controversial as most of these parameters are from the pre-molecular era [38,39]. Therefore, the simple use of an age cut-off at 40 years, knowing the strong association between the status of *IDH*-mutation and age, is therefore outdated [19,40]. Furthermore, we observed that photon radiotherapy basically replaced by proton radiotherapy, which could be explained by an intention to avoid radiotherapy induced side effects [41].

Long-term follow-up is needed to look more closely at the tradeoff between survival benefit and functional consequences with more aggressive initial therapy. The ongoing PRO-GLIO study (NCT05190172), an RCT study on photon versus proton radiation in *IDH*-mutated LGG, is looking more closely at potential long-term adverse effects and survival benefits with different radiotherapy modalities. Further studies on the optimal timing of adjuvant oncological treatment, such as the IWOT study (NCT03763422) are important as patients with dLGG have a relatively long expected survival following diagnosis. Moreover, studies on prognostic factors stratified by the molecular subtype are needed to better define high-risk patients.

### Limitations

Limitations include those associated with a retrospective study design and the lack of assessable imaging data for all cases in the earliest period where early postoperative MRI was not a part of the standard clinical routine. Neuropsychological assessment was not routinely done in the earlier time periods with possible detection bias for cognitive and more subtle neurological problems, as discussed in more detail above. The inherent small sample size limits the statistical power to detect smaller, but still meaningful differences over time.

## Conclusion

In our center, the number of resections remains stable, but a more active surgical attitude resulted in more complete resections in recent years. No apparent increase in surgical morbidity was observed over time. Adjuvant chemotherapy has been implemented as part of standard-of-care for dLGG. In our center, radiotherapy has shifted from exclusively photons to a situation where a majority of patients now receive proton radiotherapy.

## Electronic supplementary material

Below is the link to the electronic supplementary material.

Supplementary Material 1
