# Supplementary material for: Changes in clinical management of diffuse IDH-mutated lower-grade gliomas: patterns of care in a 15-year period
Source: J Neurooncol. 2022 Nov 25;160(3):535–43. doi: 10.1007/s11060-022-04136-y (PMC9758083; doi:10.1007/s11060-022-04136-y)
Supplement: Supplementary file 1 — Supplementary Material 1 [file 11060_2022_4136_MOESM1_ESM.docx]

Supplementary Tables

**Supplementary Table 1.** Patients with non-severe permanent deficits.

| Patient* | Time period | Fluent speech, but some problem with dys-/anomia | Quadrant-anopia | Fatigue / concentration difficulties | MRC 4** | MRC 5, but impact on fine motor skills / pace | Cognitive impact*** |
| --- | --- | --- | --- | --- | --- | --- | --- |
| 1 | Group 1: 2007-2011 |  |  | X |  |  |  |
| 2 |  |  | X |  |  |  |  |
| 3 |  |  |  | X |  |  |  |
| 4 |  |  |  | X |  |  |  |
| 5 |  | X |  | X |  |  |  |
| 6 |  |  |  | X | X |  |  |
| 7 |  | X |  |  |  |  |  |
| 8 |  |  |  |  | X |  |  |
| 9 | Group 2: 2012-2016 | X |  |  |  |  |  |
| 10 |  |  | X |  |  |  |  |
| 11 |  |  |  |  | X |  |  |
| 12 |  | X |  |  |  |  |  |
| 13 |  | X |  |  |  |  |  |
| 14 |  |  |  |  | X |  |  |
| 15 |  |  | X |  |  |  |  |
| 16 |  | X |  | X |  |  |  |
| 17 |  | X |  |  |  |  |  |
| 18 |  | X |  |  |  |  | X |
| 19 |  | X |  |  |  |  |  |
| 20 |  | X |  |  |  |  |  |
| 21 | Group 3: 2017-2021 |  | X |  |  |  |  |
| 22 |  |  | X |  |  |  | X |
| 23 |  | X |  |  |  |  |  |
| 24 |  |  |  |  |  | X |  |
| 25 |  | X |  |  |  |  |  |
| 26 |  |  |  | X |  | X |  |
| 27 |  | X |  |  |  | X |  |
| 28 |  |  | X |  |  |  |  |
| 29 |  |  |  |  |  |  | X |
| 30 |  | X |  |  |  |  |  |
| 31 |  | X |  |  |  |  |  |
| 32 |  | X |  |  |  |  |  |
| 33 |  |  |  | X |  |  |  |
| 34 |  |  |  | X |  |  |  |

* One patient per row

** MRC one or more muscle groups; 0 = no contraction; 1 = flicker or trace of contract; 2 = active movement with gravity eliminated; 3 = active movement against gravity; 4 = active movement against gravity and resistance; 5 = normal power.

*** Memory, visuospatial function and/or executive function.

**Supplementary Table 2.** Patients with severe permanent deficits.

| Patient | Time period | Speech difficulties that prevent effective communication | MRC ≤ 3* | Cognitive impact** |
| --- | --- | --- | --- | --- |
| 35 | Group 1: 2007-2011 |  | X |  |
| 36 | Group 2: 2012-2016 |  | X |  |
| 37 |  | X | X |  |
| 38 |  | X | X |  |
| 39 |  |  |  | X |
| 40 |  |  | X |  |
| 41 | Group 3: 2017-2021 | X | X |  |
| 42 |  |  | X |  |
| 43 |  |  | X |  |
| 44 |  |  |  | X |

*MRC one or more muscle groups; 0 = no contraction; 1 = flicker or trace of contract; 2 = active movement with gravity eliminated; 3 = active movement against gravity; 4 = active

**memory, visuospatial function and/or executive function.

**Supplementary table 3.** Table showing segmentation volumes, complete resection, surgical and oncological treatment for astrocytomas grade 2 and 3, n=109

|  | Group 1:  2007-2011  n = 35 | Group 2:  2012-2016  n = 41 | Group 3:  2017-2021  n = 33 | P-value |
| --- | --- | --- | --- | --- |
| **Segmentation volume** | | | | |
| Preoperative (mL), median (Q1, Q3)* | 69.0 (38.6, 130.8) | 59.4 (32.9, 108.7) | 45.2 (25.1, 102.2) | 0.449 |
| Postoperative (mL), median (Q1, Q3)* | N=17  5.7 (2.0, 70.4) | N=40  13.2 (2.0, 49.6) | N=33  5.0 (0.7, 18.0) | 0.356 |
| Gross total resection, n (%) | N=31  2 (6.5) | N=40  7 (17.5) | N=33  7 (21.2) | 0.235 |
| **Surgical treatment** | | | | |
| Resection, n (%)  Biopsy, n (%) | 33 (94.3)  2 (5.7) | 39 (95.1)  2 (4.9) | 31 (93.9)  2 (6.1) | 0.97 |
| **Oncological treatment** | | | | |
| Only chemotherapy^1^, n (%) | 1 (2.9) | 4 (9.8) | 7 (21.2) | 0.051 |
| Only radiotherapy^1^, n (%) | 11 (31.4) | 5 (12.2) | 4 (12.1) | 0.053 |
| Concomitant chemoradiotherapy^2^, n (%) | 11 (31.4) | 21 (51.2) | 10 (30.3) | 0.107 |
| Sequential chemoradiotherapy^2^, n (%) | 0 (0.0) | 4 (9.8) | 13 (39.4) | **<0.001** |
| No adjuvant treatment^3^, n (%) | 12 (34.3) | 7 (17.1) | 5 (15.2) | 0.102 |

^1^Started within 6 months after surgery (see details under Methods).

^2^Within 12 months after surgery. Only TMZ was administered concomitant with radiotherapy.

^3^Within 6 months after surgery.

*Indicates that values are missing, see actual N provided per cell.

**Supplementary table 4.** Table showing segmentation volumes, complete resection, surgical and oncological treatment for oligodendrogliomas grade 2 and 3, n=93.

|  | Group 1:  2007-2011  n = 26 | Group 2:  2012-2016  n = 31 | Group 3:  2017-2021  n = 36 | P-value |
| --- | --- | --- | --- | --- |
| **Segmentation volume** | | | | |
| Preoperative (mL), median (Q1, Q3)* | N=25  42.1 (20.1, 113.0) | N=30  57.7 (28.4, 85.3) | N=36  44.5 (21.9, 81.9) | 0.91 |
| Postoperative (mL), median (Q1, Q3)* | N=13  9.5 (3.0, 39.3) | N=30  8.5 (1.6, 16.5) | N=36  3.3 (0.0, 25.0) | 0.91 |
| Gross total resection, n (%) | N=18  1 (5.6) | N=30  4 (4.8) | N=36  11 (30.6) | 0.05 |
| **Surgical treatment** | | | | |
| Resection, n (%)  Biopsy, n (%) | 25 (96.2)  1 (3.8) | 30 (92.8)  1 (3.2) | 35 (97.2)  1 (2.8) | 0.97 |
| **Oncological treatment** | | | | |
| Only chemotherapy^1^, n (%) | 0 (0.0) | 9 (29.0) | 8 (22.2) | **0.014** |
| Only radiotherapy^1^, n (%) | 9 (34.6) | 1 (3.2) | 4 (11.1) | **0.003** |
| Concomitant chemoradiotherapy^2^, n (%) | 3 (11.5) | 6 (19.4) | 1 (2.8) | 0.091 |
| Sequential chemoradiotherapy^2^, n (%) | 0 (0.0) | 9 (29.0) | 22 (61.1) | **<0.001** |
| No adjuvant treatment^3^, n (%) | 14 (53.8) | 10 (32.3) | 8 (22.2) | **0.034** |

^1^Started within 6 months after surgery (see details under Methods).

^2^Within 12 months after surgery. Only TMZ was administered concomitant with radiotherapy.

^3^Within 6 months after surgery.

*Indicates that values are missing, see actual N provided per cell.
